# Transient Compression of Coronary Artery-Left Ventricular Micro-Fistulae by Stunned Myocardium in Takotsubo Syndrome

**DOI:** 10.31083/RCM52518

**Published:** 2026-10-12

**Authors:** Shams Y-Hassan

**Affiliations:** ^1^Department of Cardiology, Karolinska Institutet and Karolinska University Hospital, 141 86 Stockholm, Sweden

**Keywords:** takotsubo syndrome, myocardial stunning, microvascular dysfunction, coronary artery fistula, myocardial contraction bands

## Abstract

Takotsubo syndrome (TS) is an acute cardiac disease characterized by a unique regional, circumferential, and reversible left ventricular wall motion abnormality (LVWMA). The characteristic feature of LVWMA in TS is myocardial stunning, in which the affected myocardium represents a state of cardiac “cramp”. The severity of this myocardial cramp is variable. It may be severe enough to cause compression of both the intramyocardial coronary micro- and macrocirculation. Currently, only one case has been published featuring TS and compressed (or invisible) coronary artery-left ventricular micro-fistulae (CALVMF) during the acute stage of TS, along with its reappearance as the left ventricular function normalized. In this report, evidence for complete compression of CALVMF arising from both the left anterior descending artery and posterior descending artery draining into the left ventricle during the acute stage of TS in a second case is presented. This observation was confirmed by the reappearance of CALVMF on follow-up coronary angiography after left ventricular function had completely normalized. This novel finding further indicates that myocardial stunning in TS represents a state of cardiac cramp and that the coronary microvascular dysfunction observed in some patients with TS is secondary to compression of the intramyocardial resistance microcirculation.

## 1. Introduction

Takotsubo syndrome (TS) is an acute cardiac disease with a clinical presentation resembling that of an acute coronary syndrome [1]. The disease is characterized by a unique regional, circumferential, and usually reversible left (and in some cases right) ventricular wall motion abnormality (LVWMA) [1,2]. The LVWMA in TS has the characteristic features of myocardial stunning [3,4]. The cardiac imaging characteristics of LVWMA and the histopathological features of myocardial stunning in TS indicate that the affected myocardium is in a state of myocardial cramp (a rigid cramp-like state of the heart muscles) [3,4]. Studies have demonstrated that this myocardial cramp causes compression of the resistance intramyocardial microcirculation, resulting in coronary microvascular dysfunction (CMVD) in some patients with TS [3,4]. The myocardial stunning can also cause transient compression of the conductance intramyocardial coronary macrocirculation involving the septal branches arising from the left anterior descending artery (LAD), the coronary artery segments affected by myocardial bridging, as well as the epicardial coronary arteries, especially the LAD [4]. In this report, we provide evidence and illustrative cardiac images to show that myocardial stunning in TS causes transient compression of the coronary artery-left ventricular micro-fistulae (CALVMF).

## 2. The Stunned Myocardial Segments in TS Are in a State of Cardiac “Cramp”

The LVWMA in TS exhibits all the characteristic features of myocardial stunning [3,4]. The sharp transition at the hinge point between the akinetic segments of the left ventricle and the valve-like motion of the hyperkinetic basal segments of the left ventricle in the mid-apical pattern of TS or the slingshot-like hyperkinetic apical segments of the left ventricle in mid-ventricular or basal patterns of TS indicates that the akinetic segments are stiff [3,4]. Some researchers [5] hypothesize that these extreme and opposing wall motion changes in TS (akinesia and hyperkinesis) cause a hinge-point distortion of the coronary arteries, triggering spontaneous coronary artery dissection, and have named this phenomenon “acute mechano-cardiac coronary artery disruption” [5]. The LVWMA and myocardial deformation analysis in TS seen on echocardiography-2D-strain imaging has shown findings indicating uniform myocardial shortening despite the akinetic appearance of the left ventricular apex [6]. There were also findings indicating that the LVWMA could be the result of left ventricular geometry-induced regional differences in wall stress [6]. Recently, left ventricular mechanics assessed by 2D-speckle-tracking echocardiography were compared between patients with TS and those with ST-elevation myocardial infarction caused by an LAD occlusion. This study revealed that global radial strain was more significantly impaired in patients with TS. This finding is consistent with the diffuse stress-induced myocardial stunning seen in patients with TS [7].

The cramp-like cardiac imaging of the hypo- and akinetic segments of the myocardium in TS is confirmed by the myocardial histopathological findings in TS, where important and consistent changes include hypercontracted sarcomeres and contraction bands [8], as well as the autopsy findings of a stiffened myocardium, which has lost its pliability [9]. All these changes in the akinetic segments of the myocardium indicate that the affected cardiac muscles in the acute stage of TS are in a state of “cramp”.

## 3. Myocardial Stunning Induces Transient Compression of the Intramyocardial Coronary Micro- and Macrocirculation

Invasive and non-invasive studies of the coronary microcirculation have shown signs of reversible CMVD in some patients with TS [10,11,12,13,14,15]. Furthermore, nuclear myocardial perfusion imaging studies displayed nuclear stress defects corresponding to the segments with LVWMA in patients with TS and non-obstructive coronary arteries [16,17]. In a recent study [18], serial positron emission tomography perfusion imaging in 62 patients with TS revealed reversible reductions in rest and stress myocardial blood flow and myocardial flow reserve as well as increases in coronary vascular resistance, suggestive of TS-related CMVD, which extended beyond regions of wall motion abnormalities. Furthermore, the regional coronary flow abnormalities persisted even after recovery of left ventricular function. These signs strengthen the evidence for CMVD in TS. For these reasons, some researchers believe that CMVD is the main cause of the pathogenesis of TS. However, evidence arguing against this hypothesis has been reported [3,4]. The CMVD is most probably a secondary phenomenon caused mainly by compression of the resistance intramyocardial circulation by the stunned myocardium. Furthermore, sufficient evidence exists that the stunned myocardium also compresses the intramyocardial conductance vessels (such as the septal branches arising from the LAD) and the coronary artery segments with myocardial bridging, particularly the LAD [3,4,19].

## 4. Transient Compression of CALVMF by the Stunned Myocardial Segments in TS

CALVMF is a relatively rare condition and may be an incidental finding during coronary angiography [20]. The association of TS and visible (non-compressed) CALVMF during the index presentation has been reported as an incidental finding [21,22,23,24]. Currently, only one case of transient compression of CALVMF (between the obtuse marginal branch and the left ventricle) during the acute stage of a mid-apical ballooning pattern of TS has been reported [4]. We now report this phenomenon of transient compression of CALVMF in an additional patient with TS during the acute presentation, in which the CALVMF subsequently reappeared during follow-up coronary angiography, and is demonstrated in illustrative cardiac images. The patient, an elderly female with chronic obstructive pulmonary disease (COPD), presented with an acute exacerbation of COPD and chest pain. The electrocardiogram revealed sinus rhythm and left bundle branch block (Fig. 1A). There was a rise and fall of troponin levels with a max troponin of 1240 ng/L. Contrast left ventriculography exhibited a typical mid-apical, apical-tip-sparing ballooning pattern of TS. This finding was confirmed by contrast echocardiography (Fig. 1B,C,D,E). Invasive coronary angiography revealed no obstructive coronary artery disease (Fig. 1F,G), but moderate slow flow was observed, especially in the LAD, which also exhibited signs of compression of the septal branches arising from both the LAD and the posterior descending artery (PDA), and compression of the small peripheral branches from the LAD system (Figs. 2,3). No signs suggestive of CALVMF were seen during the index presentation. Due to a repeat episode of chest pain 2 months after the index presentation, a new contrast left ventriculography and coronary angiography were performed. These studies revealed complete normalization of left ventricular function with normal flow in the coronary arteries and reappearance of the septal branches from both the LAD and PDA, and the peripheral small branches from the LAD system (Figs. 2,3). There were also mild signs of CALVMF from the LAD and even the PDA to the left ventricle with slight contrast opacification of the left ventricle during left and right coronary angiography (Figs. 2,3, small yellow asterisks). During repeat left ventriculography and coronary angiography 53 months after the index presentation due to recurrent chest pain, signs of extensive CALVMF from the LAD system to the left ventricle (Fig. 2), and signs of significant CALVMF from the PDA to the left ventricle were observed (Fig. 3).

**Fig. 1.**
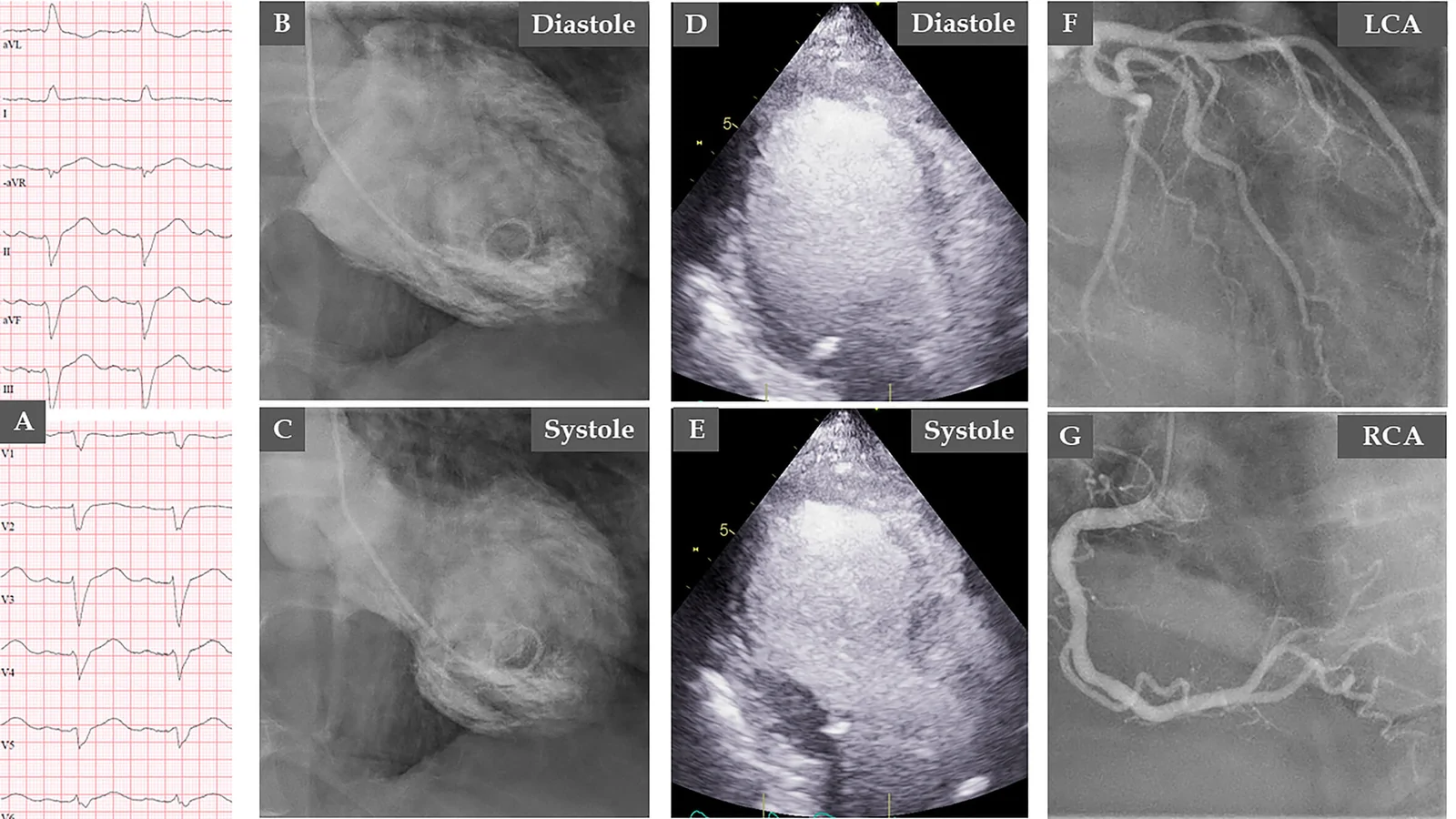
**Demonstration of a case of mid-apical with apical-tip-sparing ballooning pattern of takotsubo syndrome (TS)**. An elderly female presented with an acute exacerbation of chronic obstructive pulmonary disease. Electrocardiogram (A) shows sinus rhythm and left bundle branch block. Contrast left ventriculography during diastole (B) and systole (C) reveals a mid-apical with apical-tip-sparing pattern of TS. Contrast echocardiography during diastole (D) and systole (E) confirms the finding of left ventriculography. Left coronary angiography (LCA) in (F) and right coronary angiography (RCA) in (G) show no obstructive coronary artery lesions. Further details of LCA and RCA are described in Figs. 2,3.

**Fig. 2.**
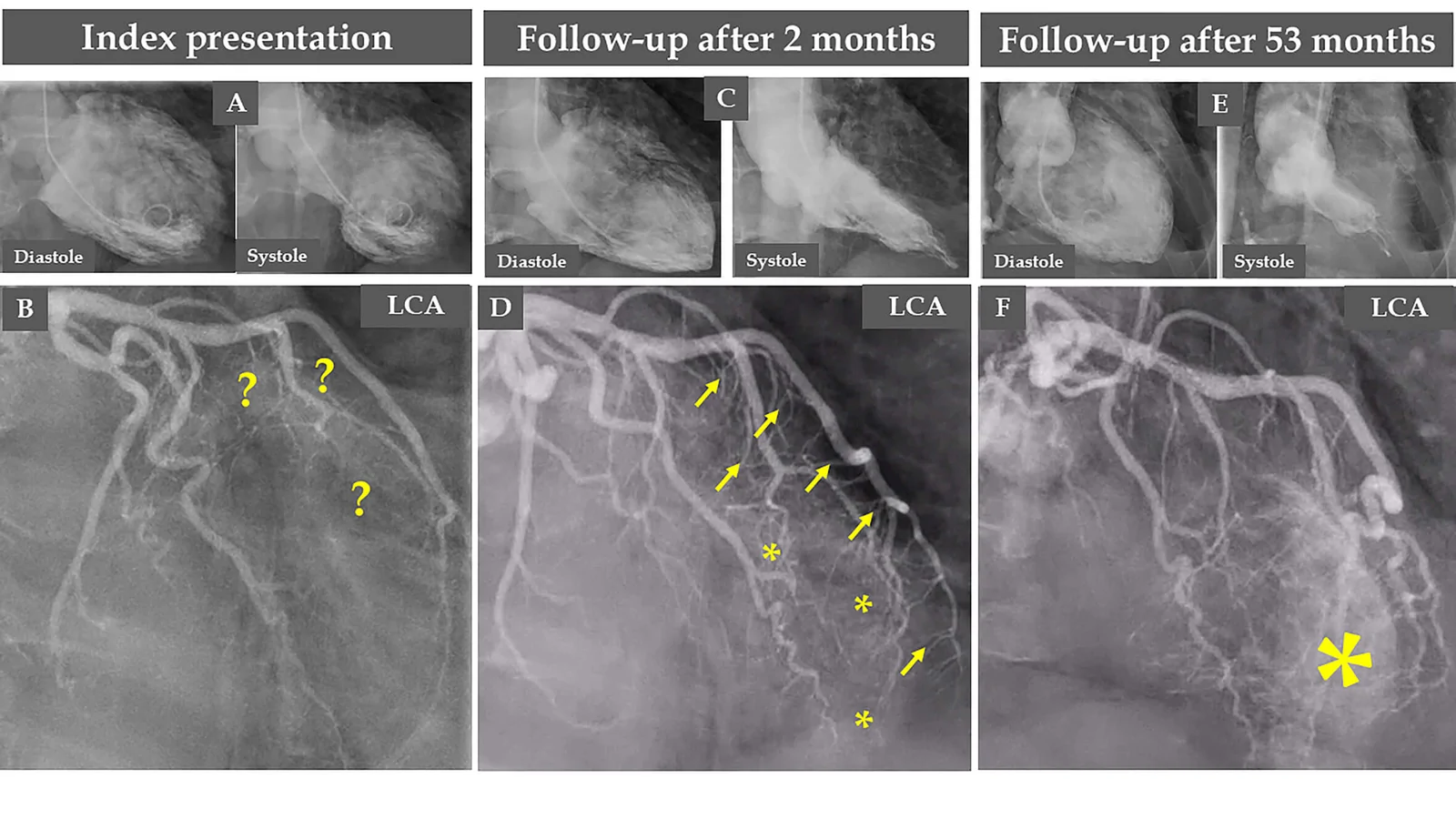
**Demonstration of left ventriculography and left coronary angiography (LCA) during the index presentation and follow-up after 2 and 53 months**. Contrast left ventriculography (A) during diastole and systole reveals a typical mid-apical with apical-tip-sparing pattern of TS during the index presentation. LCA (B) shows that flow in the left anterior descending artery (LAD) and septal branches is poor (yellow question marks). Follow-up left ventriculography (C) and LCA (D) 2 months after the index presentation show complete normalization of the left ventricular function. The LAD flow is normal with septal branches ((D), yellow arrows) and signs of coronary artery-left ventricular micro-fistulae (CALVMF) with mild contrast opacification of the left ventricle ((D), small yellow asterisks) are seen. Follow-up left ventriculography (E) and LCA (F) 53 months after the index presentation show that the left ventricular function is completely normal, and the LCA shows signs of extensive CALVMF with clear contrast opacification of the left ventricle ((F), large yellow asterisk).

**Fig. 3.**
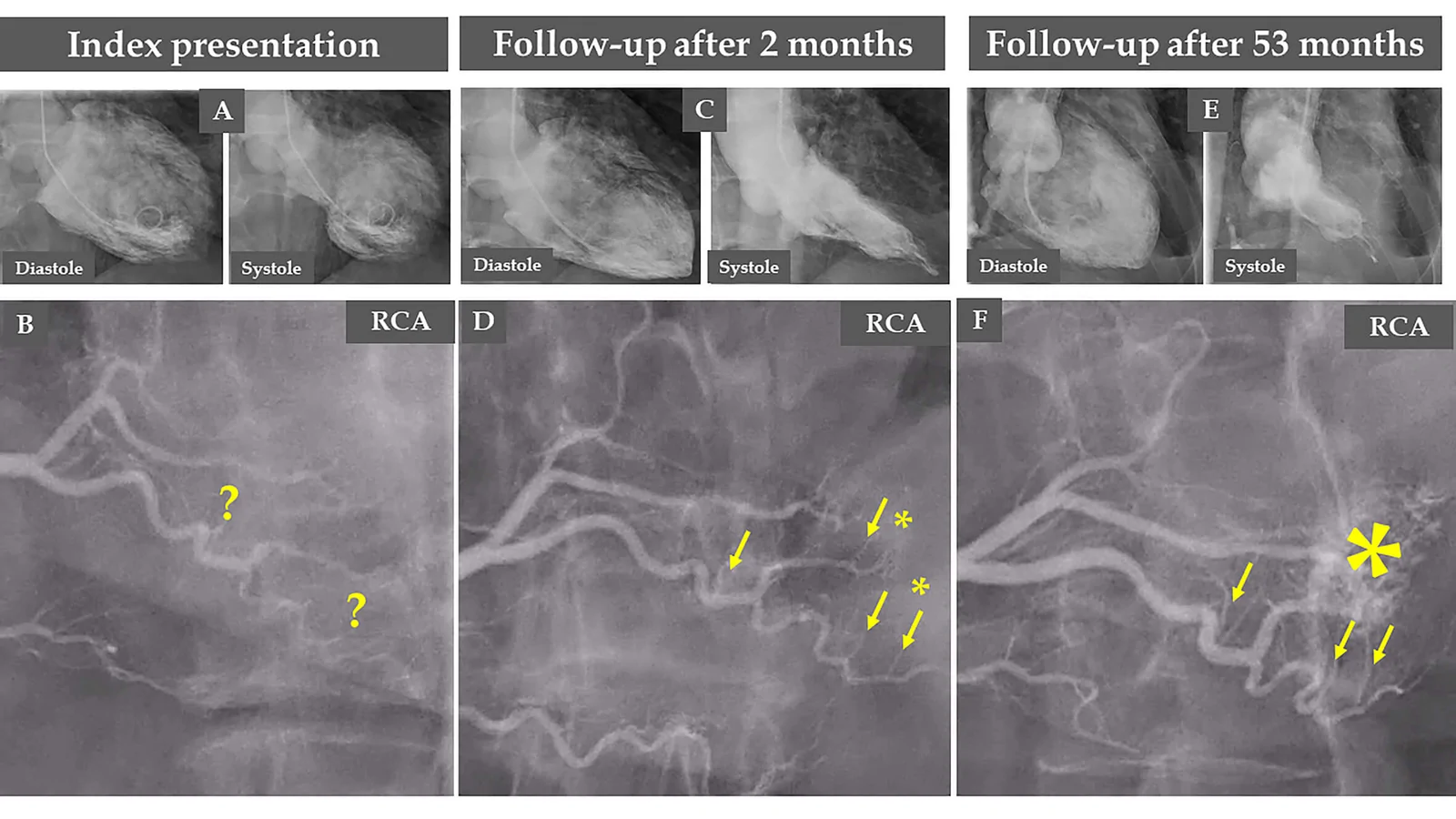
**Demonstration of left ventriculography and right coronary angiography (RCA) during the index presentation and follow-up after 2 and 53 months**. Contrast left ventriculography (A) during diastole and systole reveals a typical mid-apical with apical-tip-sparing pattern of TS during the index presentation. RCA (B) shows the peripheral branches of RCA, the posterior descending artery (PDA), and the posterior left ventricular artery. The small septal branches from the PDA are almost invisible ((B), yellow question marks). Follow-up left ventriculography (C) and RCA (D) 2 months after the index presentation show complete normalization of the left ventricular function. The septal branches from the PDA are clearly seen ((D), yellow arrows) and show certain signs of CALVMF with very mild contrast opacification of the left ventricle ((D), small yellow asterisks). Follow-up left ventriculography (E) and RCA (F) 53 months after the index presentation show that the left ventricular function is completely normal, and signs of CALVMF are clearly seen from the PDA segment ((F), large yellow asterisk) and the septal branches from the PDA are larger ((F), yellow arrows).

The recently reported case of transient compression of a CALVMF originating from an obtuse marginal branch to the left ventricle [4], and the present case of transient CALVMF compression from both the LAD and PDA during the acute stage of TS represent novel observations not previously documented by other investigators. However, this does not mean that this phenomenon has never occurred. Detecting transient CALVMF compression requires follow-up coronary angiography after the left ventricular dysfunction has fully normalized. The most plausible explanation for this is the complete compression of the micro-fistulae by the affected stunned myocardium. However, alternative explanations must be considered, such as differences in the ability to detect small fistulae on angiography, or dynamic changes in coronary tone and variability in flow conditions. The coexistence of TS and non-compressed CALVMF in a limited number of reported cases [21,22,23,24] is most probably due to mild myocardial stunning, which was not severe enough to cause complete compression of CALVMF.

## 5. Conclusions

Myocardial stunning is the characteristic feature of LVWMA in TS. Increasing evidence indicates that the stunned myocardium in TS is in a state of “myocardial cramp”, leading to transient compression of both intramyocardial coronary micro- and macrocirculation. The transient compression of CALVMF during the acute stage of TS in both the recently reported and the current case, and its reappearance during follow-up coronary angiography once left ventricular function has fully normalized, are novel observations. These findings offer further evidence that myocardial stunning in TS acts as a form of myocardial cramp. Furthermore, they argue that transient CMVD in a subset of TS patients is secondary to intramyocardial microvascular compression.

## Abbreviations

CALVMF, coronary artery-left ventricular micro-fistulae; CMVD, coronary microvascular dysfunction; COPD, chronic obstructive pulmonary disease; LAD, left anterior descending artery; LVWMA, left ventricular wall motion abnormality; PDA, posterior descending artery; TS, takotsubo syndrome.
